# *Gen1* mutation caused kidney hypoplasia and defective ureter-bladder connections in mice

**DOI:** 10.7150/ijbs.42855

**Published:** 2020-03-12

**Authors:** Xiaowen Wang, Herui Wang, Jiaojiao Liu, Yinv Gong, Chi Zhang, Francia Fang, Aiguo Li, Xiaohui Wu, Qian Shen, Hong Xu

**Affiliations:** 1 Wuhan Children's Hospital (Wuhan Maternal and Child Healthcare Hospital), Tongji Medical College, Huazhong University of Science & Technology, Wuhan 430000, China; 2 Neuro-Oncology Branch, Center for Cancer Research, National Cancer Institute, National Institutes of Health, Bethesda, Maryland, 20892, USA; 3 Shanghai Kidney Development and Pediatric Kidney Disease Research Center, Children's Hospital of Fudan University, Shanghai 201102, China; 4 Department of Molecular, Cellular, and Developmental Biology, University of Colorado, Boulder, CO 80309, USA; 5 University of Maryland School of Medicine, Baltimore, Maryland, 21201, USA; 6 State Key Laboratory of Genetic Engineering and National Center for International Research of Development and Disease, Institute of Developmental Biology and Molecular Medicine, Collaborative Innovation Center of Genetics and Development, School of Life Sciences, Fudan University, Shanghai 200433, China

## Abstract

Limited genetic factors were uncovered for the development of congenital anomalies of the kidney and urinary tract (CAKUT). We previously reported that a Holliday junction resolvase *Gen1* was essential for early metanephric development in mice. This comprehensive follow-up study focused on the roles of *Gen1* in late metanephric development. We found that *Gen1* mutation impaired the late development of both kidney and urinary tract. *In vivo* and *ex-vivo* kidney primordia culture confirmed decreased ureteric bud branching in *Gen1* mutants, which consequently caused hypoplasia. We also observed abnormal urinary tract development. Programmed apoptosis at the end of nephric duct disappeared in *Gen1* mutants, which caused abnormal ureter-bladder connections, leading to vesicoureteral reflux (VUR) or ureterovesical junction obstruction (UVJO). Mechanistically, RNA-seq analysis proved that *Gen1* mutation impaired the expression of multiple regulatory genes for the metanephric development, including *Six2*. Taken together, our study provides more insight into the roles of *Gen1* in the development of the kidney and urinary tract, which may have potential clinical significance in the treatment and/or prevention of CAKUT.

## Introduction

Congenital anomalies of the kidney and urinary tract (CAKUT) are composed of multiple malformations of the kidney (renal agenesis, duplex kidney, hydronephrosis, hypoplasia) and ureter (duplex ureter, vesicoureteric reflux (VUR), pelviureteric junction obstruction, ureterovesical junction obstruction (UVJO)). It's the most frequent congenital organ malformation (about 0.3-0.6% of all pregnancies) and accounts for more than 50% of the abdominal defects in neonates 1, 2. CAKUT is the primary cause of renal failure in infants and children 1. Children with CAKUT are also prone to hypertension and cardiovascular disease 3. Developmental defects of the kidney and urinary tract are believed to be the primary cause of CAKUT. Although the majority of CAKUT patients are sporadic, about 10% of CAKUT cases are familial with incomplete and variable penetrance 4. Specific mutations of several genes regulating kidney and urinary tract development have been reported in individual CAKUT patients 4, 5, suggesting that genetic factors play important roles in the development of CAKUT diseases.

In mammals, kidney development proceeds through three successive phases: pronephros, mesonephros, and metanephros, of which the last one develops into the final kidney. Metanephros develops from embryonic intermediate mesoderm through interactions between nephric duct and metanephric mesenchyme. In mice, the ureteric bud (UB) grows out from nephric duct around embryonic day 10.5 (E10.5) and then invades into the metanephric mesenchyme. The first T-shaped branching of ureteric bud occurs around E11.5, followed by repetitive branching and mesenchymal-to-epithelial transition. At the same time, the trunk of the ureteric bud will differentiate into ureters. Normal ureter-bladder connection occurs through programmed apoptosis of the common nephric duct (CND) 6. Mis-localized ureteric bud may lead to defective apoptosis of CND and cause VUR or UVJO 6.

The development of the kidney and urinary tract is tightly regulated by multiple genes. Currently more than 20 monogenic CAKUT-causing genes (*SIX1, SIX2, EYA1, PAX2, RET*, etc) have been identified, which have been proved to be vital for kidney and/or urinary tract development in murine models 4, 7-9. However, most of these genes were found in familial syndromic cases and could only explain a small subset of the CAKUT population. More CAKUT disease related genes and the pathological mechanisms are still in urgent need to be uncovered.

*GEN1* was identified as the first classical Holliday junction resolvase in human cells in 2008, whose essential roles in DNA damage repair in mammalian cells have been uncovered in multiple reports 10-12. However, the physiological roles of *GEN1* during development is still unclear. We previously reported that *Gen1* mutation caused CAKUT-like defects in mice, including renal agenesis, duplex kidney, and hydronephrosis 13. At early kidney development stage, compromised *Gen1* expression caused ectopic ureteric bud formation and first branching failure by disrupting GDNF signaling pathways 13. 18% of the mutant embryos suffered from renal agenesis due to the failed first branching. In the remaining 82% of the mutant embryos, it's still unclear about *Gen1* roles in later metanephros development after escaping the first branching failure.

In this study, we performed a more comprehensive follow-up analysis of the urinary system in *Gen1* mutant mice. Except for the previous reported defects, more defects were discovered, including kidney hypoplasia, VUR and UVJO. We also proved that *Gen1* regulates the expression of multiple regulatory genes for the metanephric development, including *Six2*. Our study supports the potential clinical significance of *GEN1* in the diagnosis, treatment and prevention of CAKUT.

## Results

### Disrupted *Gen1* expression in *Gen1* mutant kidney

**Figure 1:**
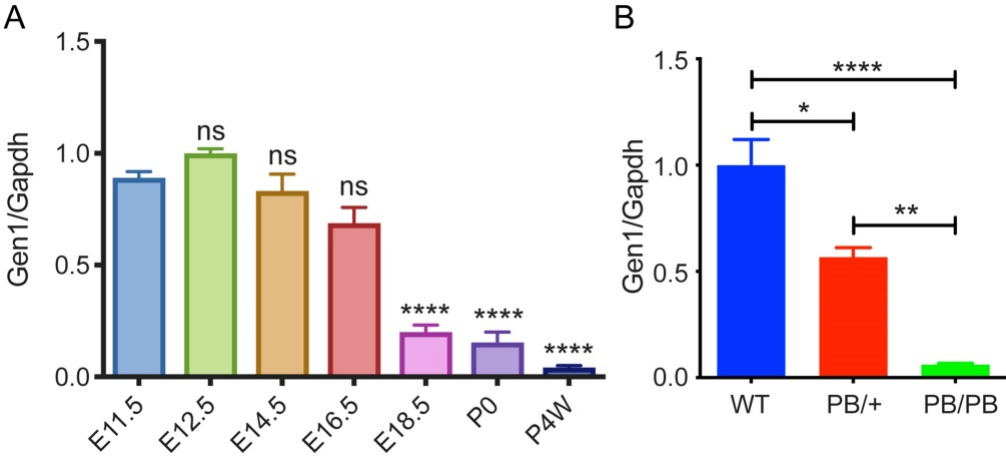
** Disrupted *Gen1* expression in *Gen1* mutant kidney. (A)** Expression level of *Gen1* in developing kidneys of wild type mice. E11.5/12.5/14.5/16.5/18.5, embryonic day 11.5/12.5/14.5/16.5/18.5; P0, postnatal day 0; P4W, 4 weeks after birth. N=3 for E11.5, E16.5, E18.5, P0, and P4W; n=8 for E12.5; n=5 for E14.5. Data was shown as mean with SEM. One-way ANOVA multiple comparisons test was performed between E11.5 and each indicated time point. **(B)** Real-time RT-PCR of *Gen1* in the E12.5 kidney primordia of wild type (WT), heterozygote (PB/+), and homozygote (PB/PB). N=4 for WT and PB/PB groups; n=3 for PB/+ group. Data was shown as mean with SEM. One-way ANOVA multiple comparisons test was performed among the three groups. ns, not significant, P>0.05; *, P<0.05; **, P<0.01; ****, P<0.0001.

We first checked the expression pattern of *Gen1* at different stages of wild type mouse kidney development. *Gen1* expression was detected in the kidney at all stages from E11.5 to weaning phase, with the highest expression in early kidney development (Fig.1A). The expression peak was detected on E12.5, and then a significant decrease on E18.5. There is still a small amount of *Gen1* expression in neonatal kidney (P0), with minimal expression four weeks after birth.

A *piggyBac* transposon (PB) insertional mutation into the second intron of mouse *Gen1* significantly disrupted its expression. We analyzed the expression of *Gen1* in the control and mutant kidney on E12.5 by real-time quantitative reverse transcription-PCR (Real-time RT-PCR). Compared to wild type kidney at E12.5, the expression level of *Gen1* in the heterozygous (PB/+) and homozygous (PB/PB) kidney decreased to 56.7% and 6.2%, respectively (Fig.1B).

### CAKUT-like defects in *Gen1* mutants

**Figure 2:**
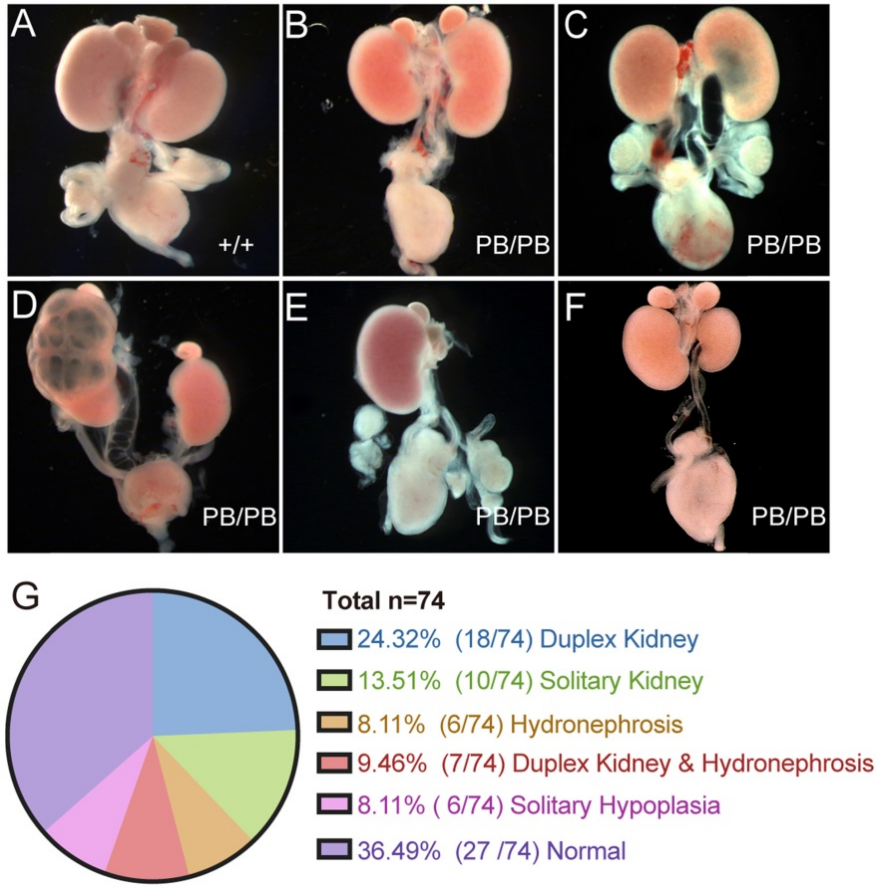
** Comprehensive summary of the kidney defects of newborn *Gen1* mutants. (A-F)** Representative images of wild type kidney (A), duplex kidney (B), hydronephrosis (C), duplex kidney and hydronephrosis (D), solitary kidney (E), and solitary renal hypoplasia (F). **(G)** Summary of the kidney symptoms in 74 *Gen1* homozygous mice.

A total of 74 newborn *Gen1^PB/PB^* mice were grossly dissected for the comprehensive analysis of the urinary system (Fig.2A-G). 63.5% (47/74) of the homozygous mutant mice showed variable CAKUT phenotypes, of which 24.3% (18/74) with duplex kidney (Fig.2B), 13.5% (10/74) with solitary kidney (Fig.2E), 8.1% (6/74) with hydronephrosis (Fig. 2C), 9.5% (7/74) with duplex kidney and renal hydronephrosis (Fig.2D), and 8.1% (6/74) with unilateral renal hypoplasia (Fig.2F). The remaining 27 mice (36.5%) were not detected for obvious CAKUT phenotypes, indicating incomplete penetrance of such *Gen1* insertional mutation in the development of urinary system. No gender or side preference was observed.

**Figure 3:**
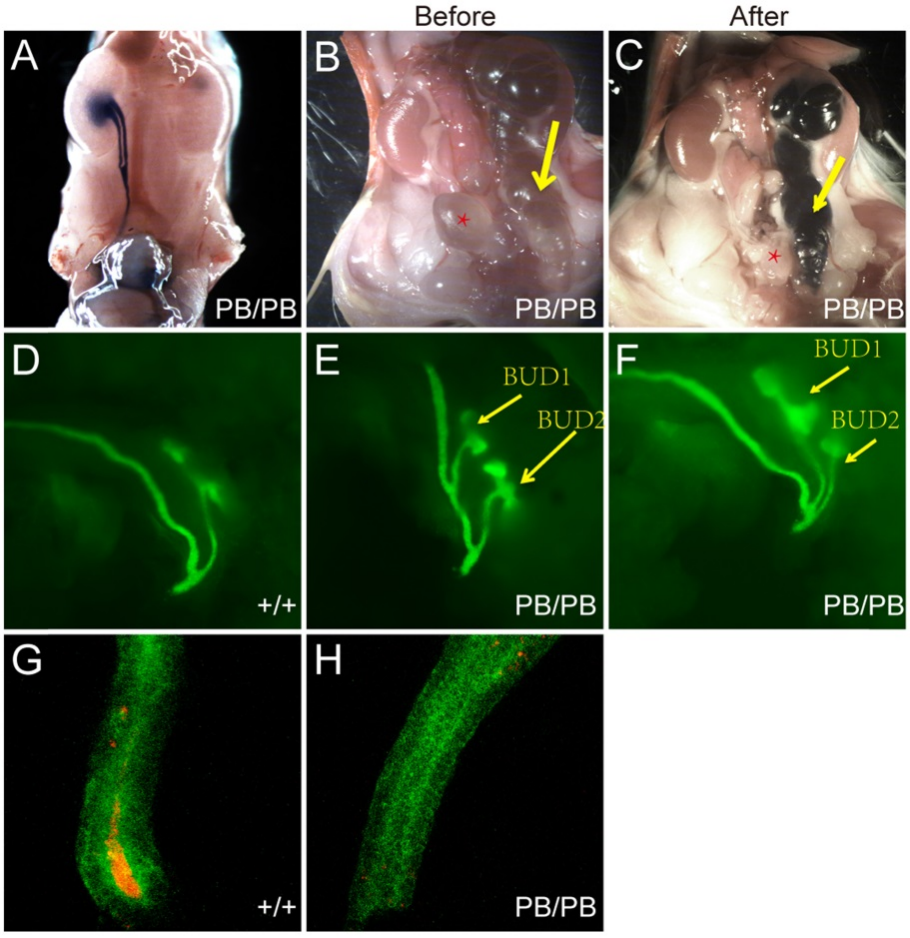
** Urinary tract defects in newborn *Gen1* mutants. (A)** Methylene blue injection in bladder revealed VUR in *Gen1* mutant. **(B)** Hydroureter in one *Gen1* mutant. Yellow arrow, hydroureter; star, bladder. **(C)** Methylene blue injection into the renal pelvis of the same mouse as in (B) revealed UVJO defect in *Gen1* mutant. **(D)** T-shaped UB branch of wild type embryo at E11.5. **(E-F)** Two UBs formed from one nephric duct in *Gen1* mutants. The anterior bud was distant from the posterior bud in (E), and adjacent to the posterior bud in (F). **(G-H)** Anti-Caspase3 staining in wild type (G) and *Gen1* mutant common nephric duct (CND).

We also checked the urinary tract defects in 29 newborn* Gen1^PB/PB^* mice and confirmed VUR in five mutant mice (Fig.3A). No reflux was observed in all of 15 wild-type newborn control mice. In the mutant mice with VUR, methylene blue refluxed to the renal pelvis, and no significant ureteral or renal pelvic dilatation were observed. All mice with reflux were combined with kidney abnormalities: four with duplex kidneys, and one with renal hypoplasia.

For *Gen1* mutant mice with hydroureter, we injected methylene blue into the pelvis and check whether the hydronephrosis was caused by obstruction of the ureteropelvic junction or the ureterovesical junction. The results showed that the methylene blue could not flow into the bladder, suggesting that the hydronephrosis was caused by the ureterovesical junction obstruction (UVJO) (Fig.3B,C). Some of the ureters were blind and did not connect to the bladder.

Mis-localized ureteric bud (UB) was reported to be causative of the abnormal ureterovesical junctions 6. To visualize the UB morphology in *Gen1* mutant mice, we introduced *Gen1* mutation in Hoxb7- mVenus transgenic mice by breeding, in which the UB and nephric ducts are positive for green fluorescence 14. We observed that UB formed in all *Gen1* mutant mice at E10.5, 59.3% (19/32) of which had ectopic UB. The ectopic budding is more clearly observed at E11.5 when the first T shaped branch occurred (Fig.3D-F). The ectopic bud was either very distant from or adjacent to the original bud (Fig.3E,F). Since mis-localized ureteric bud may lead to defective apoptosis of CND and cause VUR or UVJO, we also compared the apoptotic cells in the CND and observed much fewer caspase3 positive apoptotic cells in *Gen1* mutant CND (Fig.3G,H), which may consequently cause abnormal ureterovesical junction.

### Impaired UB branching in *Gen1* mutant mice

**Figure 4:**
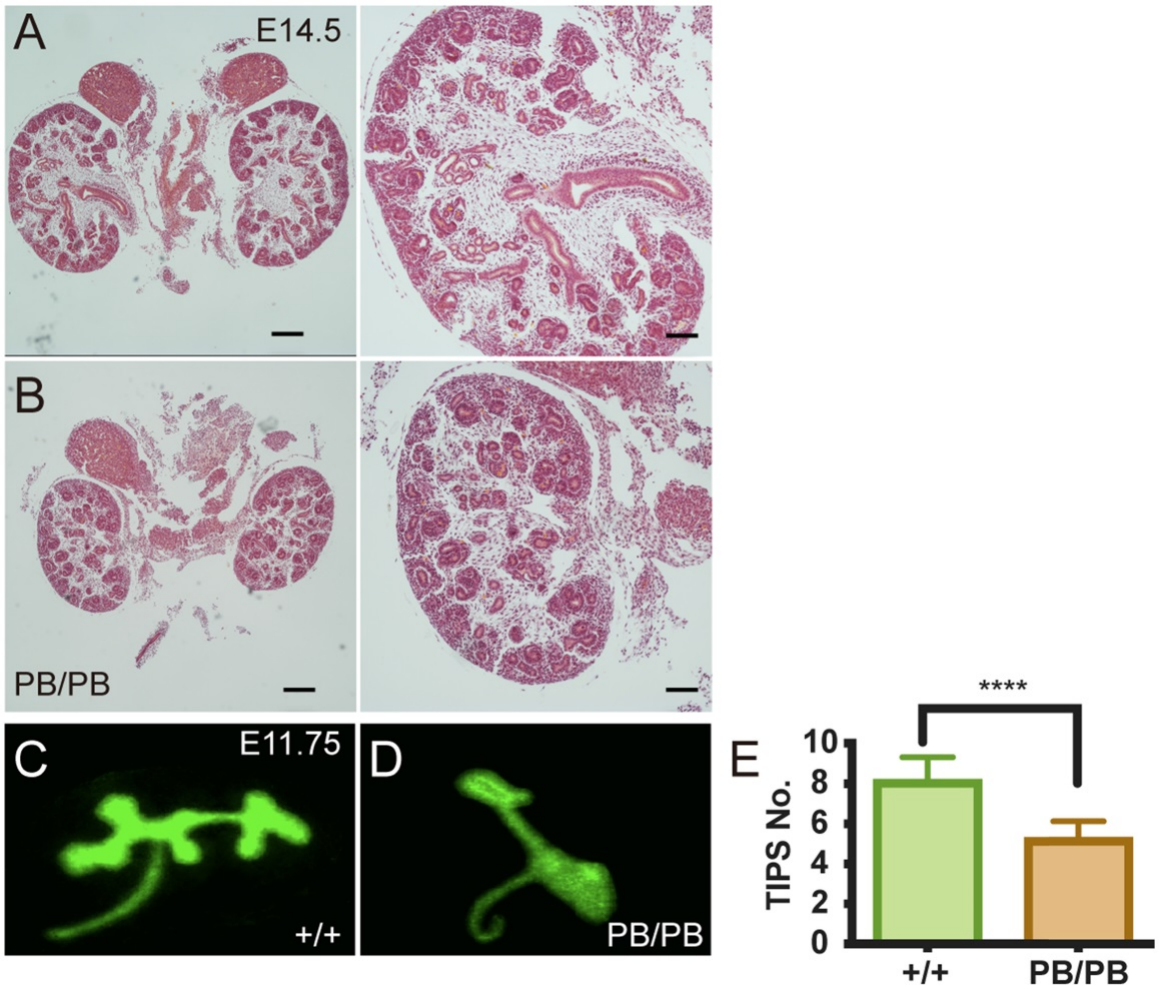
** Decreased UB branching in *Gen1* mutants. (A-B)** *Gen1* mutant kidney (B) was smaller than wild type control (A) at E14.5. Scale bars: left, 200 µm, right, 80 µm. **(C-D)** Representative images of UB branches in wild type (C) and *Gen1* mutant mice (D) at E11.75. **(E)** UB tip number in wild type and *Gen1* mutant mice at E12.5. Data was shown as mean with SD. N=10 for each group. Student's t test was performed. ****, P<0.0001.

We then investigated how loss of *Gen1* expression caused renal hypoplasia in 8.1% of the mutant mice. At E14.5, we noticed that the kidneys of some *Gen1* homozygous mice were significantly smaller than the littermate control (Fig.4A,B). There were fewer UBs and immature nephrons in the mutant kidney, indicating that the following UB branching after the first T shaped branch was impaired in *Gen1* mutants. We then checked the ureteral bud branches in the wild-type and homozygous mutants at E11.75 and E12.5 and confirmed that the number of UB branches was significantly smaller in homozygous mutant mice (Fig.4C-E).

**Figure 5:**
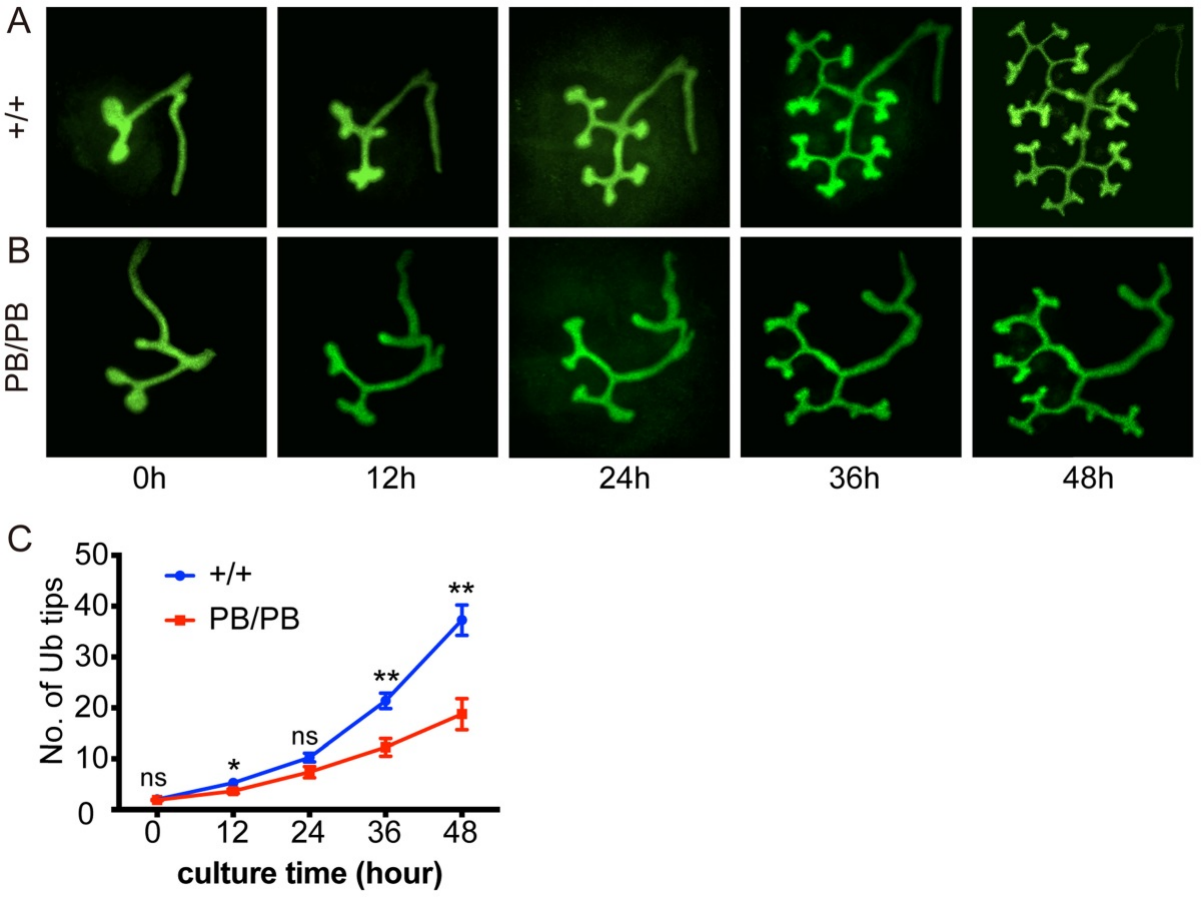
** Decreased UB branching in *ex vivo* cultured *Gen1* mutant kidney primordia. (A-B)** *Ex vivo* culture of kidney primordia isolated from E11.5 wild type (A) and *Gen1* mutant embryos (B). **(C)** Summary of the UB tip number at different observation time points. Data was shown as mean with SEM. N=8 for each group. Two-way ANOVA multiple comparisons test was performed. Ns, P>0.05; *, P<0.05; **, P<0.01.

To further confirm the effect of *Gen1* mutation on UB branching, we isolated and cultured both wild type and mutant kidney primordia at E11.5 to monitor the UB branching at different time points under the same culture condition (Fig.5A,B). The counting results also showed that the UB branch number in homozygotes was significantly smaller than that of wild type mice (Fig.5C), confirming that *Gen1* mutation impaired ureteral bud branching.

### Impaired kidney regulatory gene expression in *Gen1* mutant mice

**Figure 6:**
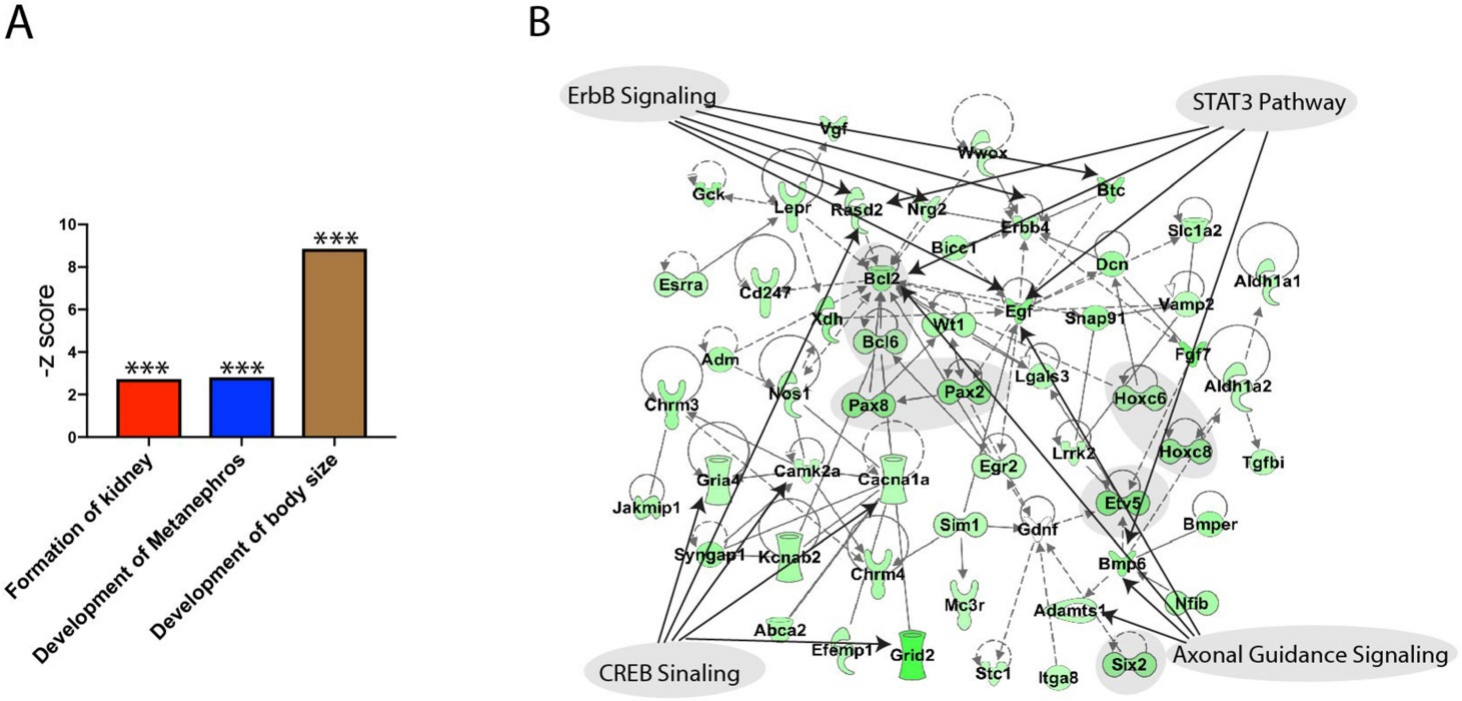
** Functional annotation of the down regulated genes by *Gen1* mutation. (A)** Three kidney development related gene sets were significantly down regulated by *Gen1* mutation. **(B)** The network built using the genes from the three significantly down regulated gene sets and the top signaling pathways mapped to it.

We performed RNA-seq to investigate the affected genes for metanephric development. RNA from E10.5 wild type and *Gen1* mutant embryo kidney primordia was extracted for RNA-seq analysis. The functional analysis revealed three significantly down regulated gene sets including the formation of kidney, development of metanephros and the development of body size, confirming the important roles of *Gen1* in kidney development (Figure 6A). We then carried out a network analysis using IPA knowledge database and build a network (Fig. 6B). The network analysis revealed that the major down-regulated genes involved in the deformed development of the mice are Pax2/8, Hoxc6/8, Bcl6, Etv5 and Six2 as shaded in the network. The top pathways that are mapped to this network are axonal guidance signaling (Adamts1, Bmp6, Egf and Bcl), ErbB signaling (Rasd2, Nrg2, Erbb4, Egf, Btc), CREB signaling (Rasd2, Camk2a, Cacna1a, Gria4, Grid2), and STAT3 pathway (Bmp6, Egf, Bcl2, Rasd2).

**Figure 7:**
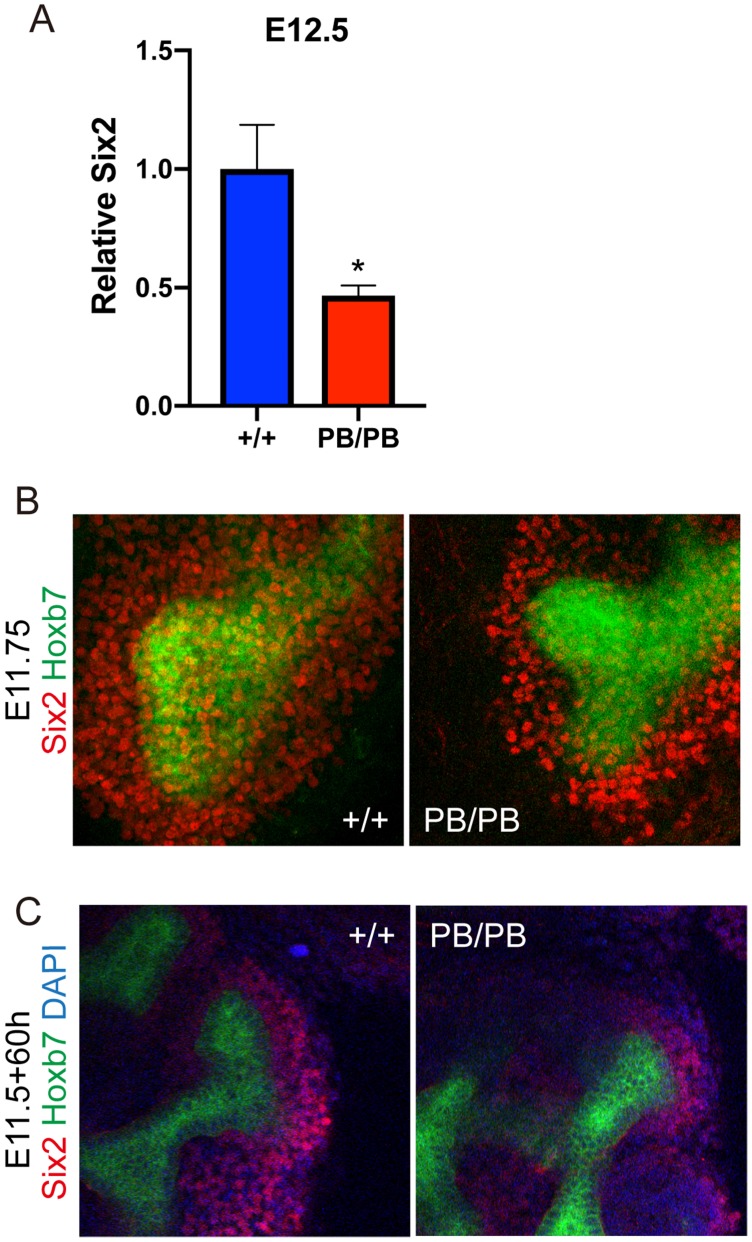
** Decreased *Six2* positive cap mesenchyme cells in *Gen1* mutant kidney. (A)** Real-time RT-PCR of *Six2* in wild type and mutant kidney primordia. Data was shown as mean with SEM. N=4 for each group. Student's t test was performed. *, P<0.05. **(B)** Immunofluorescent staining of SIX2 (red) in E11.75 wild type (left) and mutant (right) embryos. Green signal indicates the ureteric bud. **(C)** Immunofluorescent staining of SIX2 (red) in *ex vivo* cultured kidney primordia.

*Six2* promotes UB branch formation through its downstream GDNF signaling during UB branching and was one of the major affected regulator genes in the above RNA-seq results. We confirmed the decreased *Six2* expression in *Gen1* mutant kidney primordia at E12.5 by real-time RT-PCR (Fig.7A). The results showed that the expression level of *Six2* in the homozygous mutant mice was decreased to 47% of that in the wild type control (Fig.7A). We further performed whole mount immunofluorescence staining of SIX2 on E11.75 kidney primordia, which exhibited much less SIX2 positive cells surrounding the UB tip in *Gen1* mutants (Fig.7B), indicating that the cap mesenchyme layer around UB tip was much thinner in *Gen1* mutants. We also performed SIX2 staining 60 hours after *ex vivo* culture, and again observed much less SIX2 positive cells around UB tip in *Gen1* mutant primordia (Fig.7C). The smaller cap mesenchyme is likely the reason for decreased UB branching in *Gen1* mutants.

## Discussion

This study reports that *Gen1* mutant mice exhibit multiple CAKUT symptoms including solitary kidney, duplex kidney, renal hypoplasia, hydronephrosis, VUR and UVJO. To our knowledge, this is the first mouse model in which a single gene mutation caused multiple types of CAKUT phenotypes. Since the kidney and urinary tract abnormalities exhibited by *Gen1* mutant mice covered most kinds of clinical CAKUT symptoms, the *Gen1* mutant mouse model represents a unique animal model to study CAKUT pathogenesis and makes it necessary for future human genetic analysis of *GEN1* in CAKUT patients.

VUR and UVJO defects in *Gen1* mutant mice confirmed abnormal urinary tract development. Previous studies suggest that the ureteric bud sprouting position is essential for the urinary tract development 6. If it's too high, the posterior end of the ureteral trunk will be unable to properly separate from the nephric duct, so that the end of the ureter cannot be connected to the bladder, leading to obstructed ureter. On the other hand, if the UB is too low, the ureter will be abnormally positioned at the opening of the bladder, which may cause VUR. Our results showed that *Gen1* mutation disrupted the tight regulation of UB formation. The ectopic bud can be either too anterior or posterior to the nephric duct end (Fig.3E and F). Such variable UB sprouting position in the *Gen1* mutants may cause VUR or UVJO.

The formation and preservation of the cap mesenchyme is critical for metanephros development 15. The cap mesenchyme is the condensation of a subset of metanephric mesenchyme cells around the UB tip after UB invades the metanephric mesenchyme around E11. The cap mesenchyme is not only essential for inducing UB branching but also the source of progenitor cells for nephron differentiation 16. RNA-seq results showed that the expression level of cap mesenchyme markers, including *Pax2*, *Six2*, and *Wt1*, was decreased in *Gen1* mutant kidney primordia (Fig.6B). SIX2 immunofluorescent staining also confirmed thinner cap mesenchyme layer around the UB tip in *Gen1* mutant kidney primordia (Fig.7B and C). These results indicate that the smaller cap mesenchyme impaired the metanephros differentiation and led to kidney hypoplasia in *Gen1* mutants. Further studies need to be performed to address whether *Gen1* plays critical roles in the formation or preservation of cap mesenchyme.

GEN1 protein contains a PIN (PilT N terminus) nuclease domain with a helical arch/clamp region and the H3TH (helix-3-turn-helix) domain, both of which are essential for Gen1 role as a classical Holliday junction resolvase. We previously reported that GEN1 protein can also bind with the transcription factor SIX1 and enhance the transcriptional activity of SIX1/EYA1 complex in early kidney development 13. RNA-SEQ results showed that multiple regulator genes of kidney development were affected in *Gen1^PB/PB^* mutant mice (Figure 6), indicating that SIX1 was not the only target of GEN1 in kidney development. Truncated GEN1 (1-585 amino acids) that contains the potent resolvase fragment can promote SIX1/EYA1 transcriptional activity as efficiently as the full-length GEN1 protein 10, 13. It's unclear whether the regulatory roles of GEN1 in kidney development share similar mechanisms as its role of Holliday junction resolvase. Nuclease-inactive form of GEN1 (E134A/E136A) lost its resolvase activity but still promoted the transcriptional activity of SIX1/EYA1 complex efficiently 13, which indicates that GEN1 regulatory roles in kidney development are independent of its resolvase activity. However, we cannot exclude the possibility that both roles of GEN1 may share certain functional domains, e.g. DNA binding domains (helical arch/clamp region and H3TH domain).

## Material and Methods

### Generation of Mutant Mice

All animal experiments were performed under the protocols approved by the Animal Care and Use Committee (ACUC) of the Institute of Developmental Biology and Molecular Medicine (IDM) at Fudan University. *Gen1* mutant (*Gen1^PB/PB^*) mice were established by PB insertional mutagenesis 13. Hoxb7/myr-Venus transgenic mice were generated with a gift plasmid from Dr. F. Costantini (Columbia University, New York, NY). All mice strains were established and maintained on an FVB/N background.

### VUR and urinary tract obstruction

Newborn mice were tested for VUR by needling the bladder and injecting methylene blue dye as previous description 13. In the test of obstruction, methylene blue dye was injected into the renal pelvis of kidneys. The passage of dye was observed to see if it can reach the bladder. The observation time was set to 5 min.

### Organ culture

E11.5 kidneys were dissected according previous method and placed on 0.4μm Transwell filter (Corning). Explants on the filters were cultured at the air-medium interface in Dulbecco's modified Eagle's medium (Gibco) supplemented with 10% fetal calf serum (Gibco) and 1% penicillin/ streptomycin solution at 37°C with 5% CO2. Explants were observed and taken imagine every 12 hours.

### Hematoxylin-eosin (H&E) staining

H&E staining was performed as previously described 17. Briefly, embryos and kidneys from newborn mice were fixed in 4% PFA in PBS overnight, dehydrated through graded alcohols, embedded in paraffin wax and subsequently sectioned (7μm).

### Immunofluorescent staining

The immunofluorescent staining of both cultured organ and *in vivo* organ were similar. Tissues were fixed in 4% paraformaldehyde in PBS for 20mins on ice. After soaked in PBS/1% Triton X-100 for 20 min and rinsed with PBS several times, tissues were incubated PBS with 0.3% Triton X-100 and 5% normal donkey serum overnight at 4°C for blocking. Then tissues were incubated with primary antibodies overnight at 4°C. After washing 3 times in PBS 60 min each, the tissue was incubated with secondary antibodies overnight at 4 °C, washed, and mount with 50% glycerol. Images of stained tissue were captured using LSM 710 confocal laser scanning microscope using NIH ImageJ software. The antibodies were the following: rabbit anti-SIX2 primary antibody (Proteintech, 1:200), anti-caspase3 primary antibody (Cell signaling technology, 1:100), Cy5-conjugated donkey anti-rabbit secondary antibody (Jackson; 1:500).

### RNA-seq analysis

RNA-seq of kidney primordia was performed as previously described 13. Gene Set Enrichment Analysis (GSEA) preranked list was run on Gene Ontology database. The duplicate features in the data were removed and no-collapse parameter was set to 'no'. The enrichment score was calculated by a running sum statistic incremented by the absolute values of the ranking metric where a gene belongs to the set or decreased where a gene does not. The 1000 gene set permutation was used to assess the statistical significance of enrichment score (https://www.gsea-msigdb.org/gsea/index.jsp). Ingenuity Pathway Analysis (IPA) was used for network analysis and further functional pathway annotation. Specifically, the genes with FC >= 1.5 were selected and uploaded into IPA for IPA core analysis. Ingenuity knowledge base was used for the network and pathway annotation (www.analysis.ingenuity.com).

### Real-time RT PCR

Real-time RT PCR was performed as previously described 13. In brief, we extracted RNA from E12.5 embryos. After reverse transcription, we performed real-time PCR with Brilliant II fast SYBR Green QPCR master mix (Agilent Technologies, Cat#, 600843). *Gapdh* was used as internal control. Primers for each gene were as follows: Gapdh-F: TGTTCCTACCCCCAATGTGTCC; R: GGAGTTGCTGTTGAAGTCGCAG. Six2-F: AGTCCGACGTGATGTGAAC; R: AGAGAAATGAGAATTCAGGTGC. Gen1-F: GCCTGGAGTTGGAAAGGAACAAG; R: GGAACACACAGAGCAGTGAACCAC.
